# Hepatic portal venous gas with pneumatosis intestinalis secondary to mesenteric ischemia in elderly patients: Two case reports

**DOI:** 10.1097/MD.0000000000019810

**Published:** 2020-04-24

**Authors:** Minjia Wang, Jia Song, Shijin Gong, Yihua Yu, Weihang Hu, Yueben Wang

**Affiliations:** Department of Critical Care Medicine, Zhejiang Hospital, Hangzhou, PR China.

**Keywords:** hepatic portal venous gas, mesenteric ischemia, pneumatosis intestinalis

## Abstract

**Introduction:**

Hepatic portal venous gas (HPVG) is a rare imaging finding. When HPVG is accompanied with pneumatosis intestinalis (PI), the underlying cause is usually mesenteric ischemia with consequent intestinal necrosis. This combination of clinical conditions is associated with a poor prognosis. In this study, we present the cases of 2 elderly patients with HPVG and PI secondary to mesenteric ischemia.

**Patient concerns:**

In case 1, a 89-year-old male patient was admitted to intensive care unit with respiratory failure, On the fifth day of admission, he developed a high fever (39.5°C) and abdominal distension. In case 2, a 92-year-old male patient admitted to our intensive care unit and received mechanical ventilation due to acute respiratory failure. During the treatment, the patient developed gastrointestinal bleeding. On physical examination, abdominal bulging and tense abdominal walls were detected. Both patients underwent abdominal contrast-enhanced computed tomography, showed abundant HPVG with PI.

**Diagnoses:**

The patients were diagnosed as acute mesenteric ischemia, bowel necrosis, septic shock, multiple organ dysfunction syndrome based on computed tomography scan, abdominal signs, and laboratory tests.

**Interventions:**

Fluid resuscitation, high-dose vasopressors, and intravenous antibiotic therapy were given.

**Outcomes:**

Despite prompt treatment, the condition of both patients rapidly deteriorated, and the patients died shortly thereafter.

**Conclusion:**

Mesenteric ischemia is a clinical emergency. In patients with risk factors and abdominal signs, the clinical suspicion for this condition should be high. Although rare, both HPVG and PI are important radiological clues that usually indicate the presence of mesenteric ischemia with consequent intestinal necrosis.

## 1 Introduction

Hepatic portal venous gas (HPVG) was first described in 1955 in infants with necrotizing enterocolitis.^[1]^ This rare imaging finding is a diagnostic marker of severe abdominal disease. When HPVG is associated with pneumatosis intestinalis (PI), the cause is usually an intestinal ischemic event. HPVG associated with PI is a life-threatening condition, with a reported mortality rate of 75%.^[2]^ Here, we present 2 cases of HPVG associated with PI secondary to mesenteric ischemia in elderly patients.

## 2 Case presentation

### 2.1 Case 1

A 89-year-old man was admitted to our hospital with a 2-week history of cough and chest congestion. He also had a history of chronic obstructive pulmonary disease, coronary atherosclerotic heart disease, hypertension, hypercholesterolemia, and type 2 diabetes. Three days after admission, he developed severe carbon dioxide retention (pCO_2_, 96 mm Hg), which necessitated endotracheal intubation and mechanical ventilation. He was transferred to the intensive care unit (ICU) at this time. However, 5 days after admission, he developed a high fever (39.5°C) and abdominal distension. An abdominal examination revealed marked abdominal tension.

**Figure 1:**
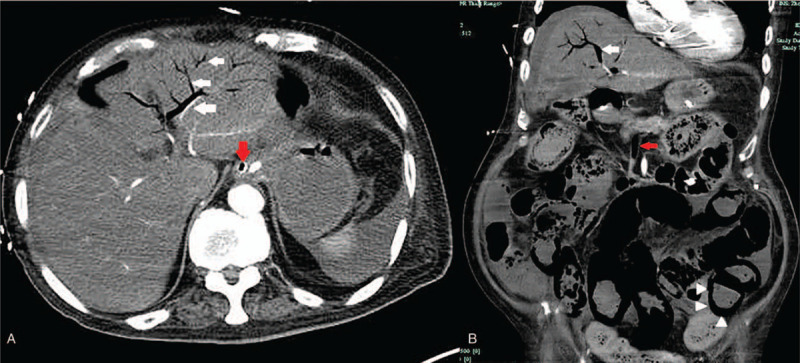
Contrast-enhanced abdominal computed tomography showing hepatic portal venous gas (A and B, white arrow) and *Pneumatosis intestinalis* (B, white triangle). Gas is also seen within the superior mesenteric veins (A and B, red arrow).

Laboratory tests revealed the following: white blood cell count, 17.1 × 10^9^/L; neutrophils, 81.8%; high-sensitivity C-reactive protein, 47.76 mg/L; and procalcitonin, 5.99 ng/mL. Arterial blood gas analysis revealed acidemia: pH, 7.30; pO_2_, 106.0 mm Hg; pCO_2_, 31.0 mm Hg; HCO_3_^−^, 15.3 mmol/L; base excess, −9.9 mmol/L; and lactate, 4.4 mmol/L. Considering the clinical presentation and examination findings, we highly suspected a diagnosis of mesenteric artery embolism. We therefore performed contrast-enhanced computed tomography (CT) of the abdomen. The abdominal CT revealed gas in the portal venous system, extending along the intrahepatic branches to within 2 cm of the liver capsule. Gas was also seen within the splenic and superior mesenteric veins. Diffuse gaseous distention of the small bowel and colon with pneumatosis of the bowel wall were present (Fig. 1). Severe atherosclerosis was detected in the mesenteric artery. A few hours after the CT examination, the patient's condition deteriorated, with loss of consciousness, hypotension (65/30 mm Hg), tachycardia (137/min), absence of urine, and other signs of septic shock. His arterial blood lactate level increased to 15 mmol/L, which was the upper limit of measurement of the blood gas analyzer (GEM Premier 3000, IL, Boston) that was used. Despite fluid resuscitation, high-dose vasopressors, and intravenous antibiotic therapy, the hypotension and anuria persisted. Due to the refractory nature of the septic shock and the high perioperative mortality risk, the consulting surgeon was of the opinion that aggressive surgical intervention would not benefit this patient. After a discussion with his family, the patient was treated palliatively. He died of multiple organ dysfunction syndrome 1 day later.

### 2.2 Case 2

A 92-year-old man was admitted to our ICU with pneumonia and acute respiratory failure, for which he underwent intubation and mechanical ventilation. A percutaneous tracheotomy was performed 3 weeks after admission due to a failure to wean from mechanical ventilation. The patient had a history of chronic kidney disease and nephrotic syndrome, for which he underwent hemodialysis four times a week. His medical history also included paroxysmal atrial fibrillation, hypertension, and Alzheimer disease. Shortly after admission to the ICU, the patient developed gastrointestinal bleeding, and a physical examination revealed abdominal distension with tense abdominal walls.

**Figure 2:**
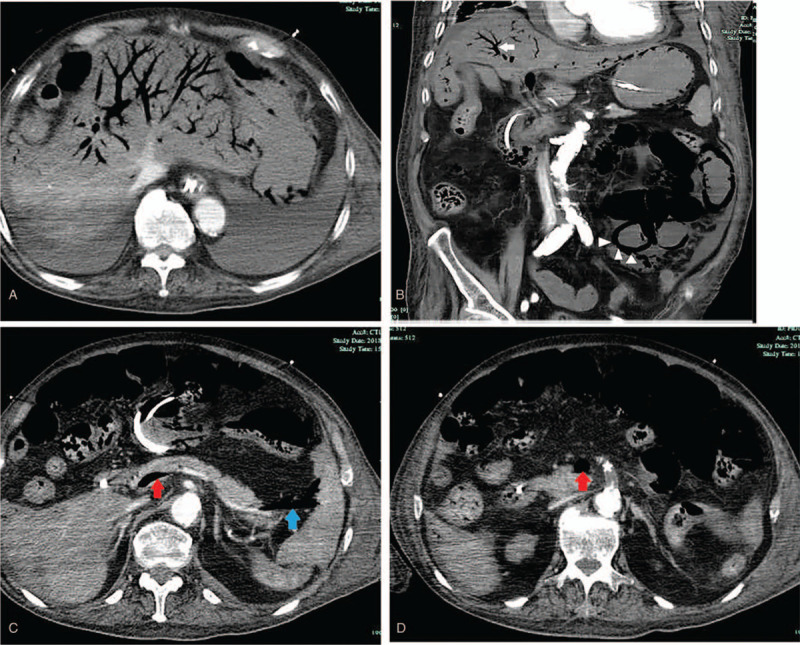
Contrast-enhanced abdominal computed tomography showing massive gas accumulation in the portal venous system and its intrahepatic branches (A) and diffuse pneumatosis intestinalis (B, white triangle). Gas is also seen throughout the superior mesenteric vein (C and D, red arrow) and splenic vein (C, blue arrow). Significant atheromatous obstruction of the ostium of the superior mesenteric artery is present (D, white asterisk).

Laboratory examinations and arterial blood gas analysis showed the following: white blood cell count, 10.7 × 10^9^/L; neutrophils, 78%; hemoglobin, 3.9 g/L; pH, 7.51; pO_2_, 210.0 mm Hg; pCO_2_, 21.0 mm Hg; HCO_3_^−^, 16.8 mmol/L; base excess, −4.3 mmol/L; and lactate, 11.7 mmol/L. Contrast-enhanced CT of the abdomen revealed an absence of parietal enhancement of the bowel, diffuse PI, and abundant HPVG. Gas was also present throughout the superior mesenteric and splenic veins. Additionally, an atheromatous obstruction was observed at the ostium of the superior mesenteric artery (Fig. 2). Refractory septic shock with disseminated intravascular coagulation precluded surgical treatment in this patient, and his condition rapidly deteriorated to multiorgan failure. He died 1 day later.

## 3 Discussion and conclusion

HPVG is defined as linear branched radiolucencies extending to within 2 cm of the periphery of the liver.^[3]^ HPVG occurs in many fatal and non-fatal conditions, such as bowel necrosis, complete or partial bowel obstruction, intraperitoneal abscess, ulcerative colitis, gastric ulcer, Crohn disease, trauma, complications of endoscopic procedures, and diverticulitis. Ischemic bowel with subsequent bowel necrosis is the primary etiology of HPVG.

In a review of 182 patients with HPVG,^[4]^ Kinoshita et al found that 43% of cases were due to bowel necrosis. The mortality rate in patients with bowel necrosis (75%) was higher than that in patients without bowel necrosis. The overall mortality rate in their cohort was 39%, which is markedly lower than the mortality rate of 75% reported by Liebman et al in 1978.^[2]^ This reduction in mortality was driven by an increase in the proportion of patients with non-fatal conditions associated with HPVG. Bowel necrosis accounted for 72% of diagnoses in the Liebman et al study.

Although the exact mechanism underlying HPVG is unknown, possible mechanisms include intestinal wall alteration, bowel distention, ischemia, and sepsis.^[5,6]^ Many diseases, including Crohn disease, ulcerative colitis, and peptic ulcer disease, as well as medical procedures such as enema and colonoscopy can result in HPVG.^[7]^ The underlying mechanisms may be related to mucosal disruption and increased intraluminal pressure, which result in the passage of air from the intestinal lumen to the mesenteric venous system. Several infectious abdominal processes have been associated with HPVG, including diverticulitis, abdominal abscess, colitis, and abdominal tuberculosis. The mechanisms involved in these settings clearly vary and may include ischemia, mucosal ulceration, and excessive gas production by invasive or luminal bacteria.^[6]^

HPVG and PI can be visualized using conventional radiography,^[5]^ CT,^[8,9]^ and ultrasonography.^[10–12]^ CT scanning is highly sensitive and is considered the gold standard imaging modality, as it offers the advantage of also detecting the associated pathology.^[13]^ The characteristic appearance of HPVG on CT imaging is a branching radiolucency extending to within 2 cm of the liver capsule, predominantly in the left lobe.^[14]^ This peripheral gas distribution is related to the direction of the blood flow in the liver. Air bubbles or continuous bands of air in the bowel wall were considered to be a sign of CT of PI.^[9]^ HPVG must be differentiated from pneumobilia, which is the presence of gas in the biliary system. Unlike HPVG, in pneumobilia, the air is located centrally and almost never extends to the periphery.^[15]^ On ultrasonography, HPVG appears as hyperechoic foci within the liver parenchyma or streak-like foci flowing within the portal veins.^[10,11]^ The advantages of ultrasonography are the rapidity of examination, low cost, possibility of bedside imaging, and the lack of radiation. The sensitivity and accuracy of ultrasonography in diagnosing HPVG are comparable to those of CT.^[11,16]^ Color Doppler flow imaging enables dynamic imaging of the centrifugal flow of portal gas to the hepatic periphery, which differentiates it from biliary gas. Both CT and ultrasonography are more sensitive than conventional radiography for diagnosing HPVG.

Historically, HPVG was an ominous finding that necessitated urgent laparotomy. In a large-scale data analysis of 1950 patients in Japan, Koizumi C et al reported a 27.3% in-hospital mortality of HPVG.^[17]^ When associated with PI, it carries a mortality rate of 75%, and thus, exploratory surgery is indicated for this condition.^[7]^ However, with the development of highly advanced imaging techniques, particularly multidetector-row CT, the detection rate of non-life-threatening cases of HPVG has increased. Because the prognosis is related to the pathology itself and is not influenced by the presence of HPVG, the treatment depends on the underlying disease and the patient's clinical condition.^[18,19]^

The 2 patients in the present study were elderly and had multiple risk factors for mesenteric ischemia, such as hypercholesterolemia, atherosclerosis, diabetes, and chronic kidney disease. They both had an acute onset with prominent abdominal signs that were highly suggestive of mesenteric ischemia. We therefore performed contrast-enhanced CT, despite the risk of renal insufficiency. The CT findings confirmed our clinical suspicion, demonstrating HPVG associated with PI, bowel distention, and severe stenosis of the mesenteric artery, which indicated acute mesenteric ischemia and bowel necrosis as the underlying etiology. According to the literature,^[7]^ emergency laparotomy is a possible option for confirmation and treatment of bowel necrosis. In the study of Koizumi C et al, 842 patients with HPVG due to bowel ischemia, 32.2% patients received operation and their in-hospital mortality was 16.5%.^[17]^ However, both our patients had a poor general condition that precluded surgery and led to a poor prognosis.

In conclusion, mesenteric ischemia is a clinical emergency. In patients with risk factors and abdominal signs, the clinical suspicion for this condition should be high. Although rare, both HPVG and PI are important radiological clues that usually indicate the presence of mesenteric ischemia with consequent intestinal necrosis. Urgent medical intervention is needed in such cases. Considering the high mortality, we believe that a general awareness of this radiological sign among clinicians may facilitate early diagnosis and intervention.

## Author contributions

Conceptualization: Min-Jia Wang, Jia Song, Shi-Jin Gong.

Data curation: Min-Jia Wang, Jia Song.

Investigation: Min-Jia Wang, Jia Song.

Methodology: Min-Jia Wang, Jia Song.

Project administration: Min-Jia Wang, Jia Song, Yue-Ben Wang.

Supervision: Shi-Jin Gong, Yi-Hua Yu.

Writing – Original Draft: Min-Jia Wang, Jia Song.

Writing – Review and Editing: Shi-Jin Gong, Yi-Hua Yu, Wei-Hang Hu, Yue-Ben Wang.

All authors read and approved the final version of the manuscript.

Shijin Gong orcid: 0000-0002-1784-2867.
